# Dysregulation of the circ_0087502/miR-1179/TGFBR2 pathway supports gemcitabine resistance in pancreatic cancer

**DOI:** 10.1080/15384047.2023.2258566

**Published:** 2023-10-16

**Authors:** Mingliu Chen, Xinxiu Liu, Jinpeng Lu, Haiwen Teng, Chengui Yu, Yingchun Liu, Yansong Zheng

**Affiliations:** a Department of hepatobiliary and pancreatic surgery, The First Affiliated Hospital of Fujian Medical University, Fuzhou, China; b Department of hepatobiliary and pancreatic surgery, National Regional Medical Center, Binhai Campus of the First Affiliated Hospital, Fujian Medical University, Fuzhou, China; c Department of ultrasound, The First Affiliated Hospital of Fujian Medical University, Fuzhou, China; d Department of Cell Biology and Genetics, Fujian Medical University, Fuzhou, China

**Keywords:** Gemcitabine resistance, circ_0087502, miR-1179, TGFBR2, pancreatic cancer

## Abstract

**Background:**

Circular RNAs (circRNAs) are a cohort of non-coding RNAs generated by back-splicing events. Accumulating evidence supports the crucial role of circRNAs in human tumorigenesis, metastasis, and chemoresistance. However, the role and mechanism of circRNA circ_0087502 in pancreatic cancer are yet unknown.

**Methods:**

The expression and function of circ_0087502 in pancreatic cancer were investigated using qRT-PCR and cell experiments. The predicted binding between circ_0087502 and microRNA-1179 (miR-1179), and between miR-1179 and TGFBR2, were examined using reporter assays.

**Results:**

Pancreatic cancer tissues and cell lines were discovered to express circ_0087502 at higher levels. Patients with pancreatic cancer who express circ_0087502 at high levels have a worse prognosis. In addition, circ_0087502 knockdown reduced the proliferation, migration, and invasion of pancreatic cancer cells and made them more sensitive to gemcitabine treatment. We found that circ_0087502 worked as a sponge for miR-1179, allowing miR-1179 to bind to the critical oncogene *TGFBR2* in its 3’-untranslated region (3’−UTR). Pancreatic cancer cells were highly resistant to gemcitabine and had increased proliferation, migration, and invasion when miR-1179 was inhibited or overexpressed.

**Conclusion:**

These results confirm that circ_0087502 activates the miR-1179/TGFBR2 axis to promote gemcitabine resistance in pancreatic cancer. Thus, our data might lay the groundwork for developing novel therapeutic strategies targeting circ_0087502 in pancreatic cancer patients.

## Introduction

Pancreatic cancer (PC) is among the top four causes of cancer death in the United States, with a 5-year survival rate of about 5% following diagnosis^1^. Surgical excision followed by adjuvant therapy with chemotherapy (including FOLFIRINOX, gemcitabine, or other drugs) is the standard of care for individuals with PC^1,2^. Unfortunately, the majority of PC patients suffer from recurring disease that is resistant to gemcitabine treatment^3^. Moreover, combination therapies produce only minor improvements in patient survival^3^. Understanding the mechanisms of resistance to anti-cancer drugs (including gemcitabine) is crucial for improving the prognosis of PC patients. Circular RNAs (circRNAs) are RNA transcripts that are produced through back-splicing and are involved in transcriptional and post-transcriptional gene regulation^4,5^.

CircRNAs have covalently closed-loop structures and are therefore more resistant to RNase R digestion than linear RNAs^4,5^. CircRNAs primarily operate as microRNA (miRNA) sponges to modulate the expression of their downstream target genes^5^. In addition, certain circRNAs have been discovered to interact with RNA-binding proteins or to serve as protein translation templates^5^. Earlier research has shown that circRNAs influence cancer biological processes, such as cell proliferation, migration, invasion, and chemoresistance^6^. For example, circ_0074298 promotes PC development and increases gemcitabine resistance by sponging miR-519^7^. However, the possible link between circRNA dysregulation and PC chemoresistance, as well as the underlying mechanisms, are unknown.

Previously, circ_0087502 was demonstrated to be highly expressed in PC tissues, and overexpression of circ_0087502 promoted cancer cell invasion and metastasis^8^. However, the effects of circ_0087502 on gemcitabine resistance in PC, and the exact underlying mechanisms are yet to be elucidated. Here, we have found that, in PC tissues, circ_0087502 expression was much higher in PC tissues than in normal tissues. By regulating the miR-1179/TGFBR2 axis, circ_0087502 was found to accelerate the proliferation, migration, invasion, and gemcitabine resistance of PC cells. Our findings show that circ_0087502 acts as a miR-1179 sponge and acts as an oncogenic circRNA in PC carcinogenesis. Thus, targeting this pathway may make PC cells more susceptible to gemcitabine therapy, and silencing of circ_0087502 might be promising for improving the effectiveness of gemcitabine in PC patients.

## Materials and methods

### Human PC specimens

The Ethics Committee of Fujian Medical University’s First Affiliated Hospital approved this work. Patients with PC at the First Affiliated Hospital of Fujian Medical University generously donated specimens (*n* = 22) collected after surgical resection. Concurrently with the removal of the tumor, adjacent normal tissues (*n* = 22) were also taken from the same patient. All individuals were found to have pancreatic ductal adenocarcinoma (PDAC) upon histological review. No preparatory care was given to these individuals before surgery. All participants completed a permission form after being told about the study’s purpose. All samples were frozen in liquid nitrogen and kept at −80°C until additional analysis could be performed.

### Cell culture

Human pancreatic cancer cell lines (PANC-1, AsPC-1, and PaCa-2) and immortalized human pancreatic duct cells (HPDE6) have been acquired from the Cell Bank of the Chinese Academy of Sciences (Shanghai, China). Dulbecco’s modified Eagle medium (DMEM, Thermo Fisher Scientific, MA, USA) was used for cultivating pancreatic cancer and HPDE6 cells. 1% penicillin-streptomycin (Sigma-Aldrich, St. Louis, MO, USA) and 10% fetal bovine serum (FBS, Thermo Fisher Scientific) were added to the medium. All cell cultures were maintained in a humidified 5% CO_2_ incubator at 37°C.

### RNA extraction and real-time PCR

TRIzol reagent (Thermo Fisher Scientific) was used to extract total RNA from the tissues and cells in accordance with the manufacturer’s protocol. A PrimeScript reagent kit (Takara, Beijing, China) was implemented to reverse-transcribe total RNA from each sample, and real-time PCR using SYBR Green (Takara) was used to amplify the resulting cDNA. NCode miRNA qRT-PCR analysis kit (Thermo Fisher Scientific) was used to measure miR-1179 expression levels. The forward (F) miR-1179 primer was designed according to the manufacturer’s instructions (Thermo Fisher Scientific), and the reverse primer (R) was the universal qPCR primer included in the kit. The relative expression levels of circ_0087502, IARS, TGFBR2 mRNA, or miR-1179 were normalized to GAPDH mRNA and U6 expression, respectively. RNase R (Sigma-Aldrich) was used to degrade circ_0087502 and its linear counterpart IARS mRNA. 2 μg of total RNA was incubated with 0.5 μl of RNase R Reaction Buffer and 0.2 μl RNase R or not for 30 minutes at 37°C. After that, the Applied Biosystems 7500 Real-Time PCR System was used to determine the relative expression levels of circ_0087502 and IARS mRNA. The follows primers were used: circ_0087502 F: 5′-AGGAACAAGGCCGACTTCTG-3′; circ_0087502 R: 5′-CCAAGATTTTCTCTTCTTCAGCAGG-3′; TGFBR2 F: 5′-GTAGCTCTGATGAGTGCAATGAC-3′; TGFBR2 R: 5′- CAGATATGGCAACTCCCAGTG-3′; GAPDH F: 5′-AATCCCATCACCATCTTC-3′; GAPDH R: 5′-AGGCTGTTGTCATACTTC-3′; U6 F: 5′-GCTTCGGCAGCACATATACTAAAAT-3′; U6 R: 5′-CGCTTCACGAATTTGCGTGTCAT-3′.

### Isolation of nuclear and cytoplasmic RNA and real-time PCR

Following the protocol provided by the manufacturer, nuclear and cytoplasmic fractions were isolated and purified using a PARIS Kit (Thermo Fisher Scientific). Expression levels of circ_0087502 were quantified in each subset using real-time PCR. GAPDH was employed as a positive control in the cytoplasm, while U6 was used as a nuclear control.

### Vectors and cell transfection

GeneChem (Shanghai, China) provided shRNA vectors that target circ_0087502 or a control shRNA vector, miR-1179 mimics, control mimics, miR-1179 inhibitor, control inhibitor, TGFBR2 expression vector, and control vector. Cells were transfected using Lipofectamine 3000 (Thermo Fisher Scientific) as per the manufacturer’s instructions. After the cells had reached 60–80% confluence, 1 μg of construct and 1 μl of Lipofectamine 3000 were added to the growth medium. Cells were harvested for analysis 48 hours after transfection. After viral infection or construct transfection, stable cell lines were identified using the appropriate antibiotics (puromycin, 1 μg/ml, Sigma-Aldrich).

### Cell proliferation assay

At a density of 3000 cells/well, cancer cells were seeded in 96-well plates and cultured for a range of times. After incubating at 37°C for 3 hours, 10 µl of Cell Counting Kit-8 reagent (Dojindo, Japan) was added. After that, we took readings of absorbance at 450 nm under various conditions.

### Wound-healing assay

In 24-well plates, cells were seeded to develop in a monolayer for 24 hours. Then, holding a sterile 200 μl pipette tip was used to scratch in each well. The unattached cells were removed by washing the plates with 500 μl PBS. After that, a fresh medium with Mitomycin C (5 μg/ml, Sigma-Aldrich) was added and incubated for 48 hours. The scratch closure was photographed under a microscope (Nikon, Japan).

### Invasion assay

When cancer cells had been planted in serum-free DMEM medium in the top chamber of Transwell chamber filters covered with Matrigel (Corning, NY, USA), they grew in a dish. Culture medium containing 10% FBS were introduced to the bottom chamber as a chemoattractant. After incubation at 37°C for 24 hours, the cells in the top chamber were discarded. After 30 minutes of staining with 0.2% crystal violet (Sigma-Aldrich) and counting using an inverted microscope (Nikon, Japan), cells that had migrated to the bottom chamber were preserved in 4% paraformaldehyde (Sigma-Aldrich).

### Cell viability assay

Cell survival was measured using the CCK-8 assessment. To test the effects of different concentrations of Gemcitabine (0–150 nM, Sigma-Aldrich), the cells were plated in 96-well plates. A CCK-8 solution (10 µl) was added to each well after 24 hours, and the plate was incubated for another 3 hours. Microplate reader (Molecular Devices, CA, USA) absorbance readings were taken at 450 nm.

### Animal experiments

The First Affiliated Hospital of Fujian Medical University’s Animal Experimental Ethics Committee authorized all animal studies before they were conducted. We ordered nude BALB/c mice from Beijing Vital River Laboratory Animal Technology in Beijing, China, when the mice were 4 weeks old. To generate a tumor xenograft model, PaCa-2 cells (5 × 10^6^) transduced with shRNA vectors targeting circ_0087502 (sh-circ_0087502) or a control shRNA vector (sh-NC) were implanted subcutaneously. Using the formula (0.5 × length × width^2^), the size of the subcutaneous tumors in the nude mice was calculated. On day 25, the tumors were removed from the naked mice and their weights recorded.

### Western blotting

RIPA lysis buffer (Thermo Fisher Scientific) was used to extract proteins from tissues and cells. Each sample’s protein content was calculated using a BCA Protein Assay Kit (Thermo Fisher Scientific). Electrophoretically, identical protein samples were separated on SDS-polyacrylamide gels. Proteins were transferred from gels to PVDF membranes (Sigma-Aldrich). The membranes were blocked at room temperature, then incubated with primary antibodies at 4°C. For a further hour at room temperature, membranes were treated with secondary antibodies. Signals were detected using an ImageQuant LAS 4000 small biomolecular imager (GE Healthcare Life Sciences, USA) and a standard ECL kit (Millipore, Billerica, MA, USA). Anti-TGFBR2 (1:1000) and anti-actin (1:5000) primary antibodies were obtained from Cell Signaling Technology (MA, USA).

### RNA immunoprecipitation (RIP) and dual-luciferase assay

PC cells were subjected to RIP tests using a RIP Kit (Millipore, Billerica, MA) following the manufacturer’s recommendations. Downstream of the luciferase reporter vector (RiboBio, Guangzhou, China), we cloned the wild-type or mutant miR-1179 binding areas in the 3’-UTR of TGFBR2 or circ_0087502 sequence. After cancer cells were implanted in 24-well culture plates for a night, they were transfected with luciferase constructs using Lipofectamine 3000 (Thermo Fisher Scientific) and miR-1179 mimics or control mimics. After 48 hours, cells were collected in Reporter Lysis Buffer from a commercial kit (Promega, Madison, WI, USA) and relative luciferase activity were determined.

### Statistical analysis

The data was presented in a mean ± SD. At least triplicates of each experiment were conducted, and all experiments were conducted at least three times. Student’s *t*-tests and one-way ANOVA analysis were used to determine statistical significance between groups. *P* values ˂ 0.05 were considered statistically significant.

## Results

### Circ_0087502, a novel circRNA, is upregulated in PC and correlated with a worse prognosis

**Figure 1.**
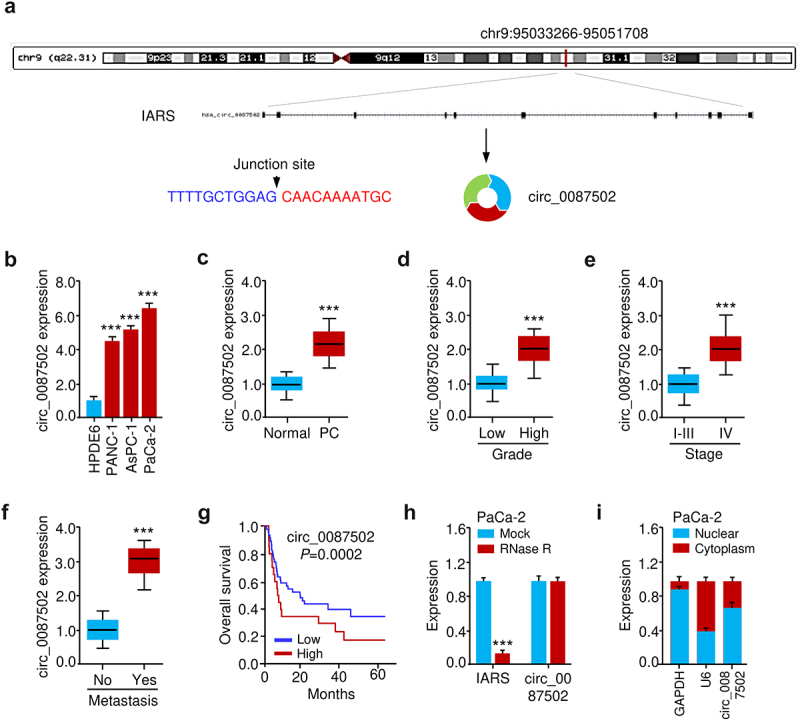
Circ_0087502, a novel circRNA, is upregulated in PC and correlated with a worse prognosis.

We showed that circ_0087502 was produced through intron-exonic back splicing of its host IARS gene, which was located on chr9 (chr9:95033266–95051708) and had a length of 1212 bp, using information from circBase, and circRNADB databases (Figure 1a). In real-time PCR assays (qRT-PCR), divergent primers for circ_0087502 were created. Human pancreatic cancer (PC) cell lines (PANC-1, AsPC-1, and PaCa-2) and immortalized human pancreatic duct cells (HPDE6) were studied for their circ_0087502 expression levels. Compared to HPDE6 cells, PC cells had significantly greater circ_0087502 levels (Figure 1b). As shown in Figure 1c, we also found that the expression of circ_0087502 was much higher in PC tissues than in adjacent normal tissues. A greater histological grade, later tumor stage, and lymph node metastasis were all associated with increased circ_0087502 expression in PC tissues (Figure 1d–f). In addition, Kaplan-Meier analysis showed that high circ_0087502 expression in PC patients was associated with a shorter overall survival time (Figure 1g). Unlike the linear IARS mRNA, which was significantly affected by RNase R treatment, circ_0087502 was rather stable after RNase R treatment (Figure 1h). Experiments separating the nucleus and cytoplasm of PaCa-2 cells showed that circ_0087502 was mostly localized in the cytoplasm (Figure 1). These data suggested that circ_0087502 is a circRNA that may promote tumor growth.

(a) It was discovered that circ_0087502 is made up of the IARS exons (chr9:95033266 -95,051,708). (b) Expression of circ_0087502 was compared between PC and control cells using quantitative real-time PCR. (c) The expression of circ_0087502 was much greater in PC tissues than in controls. Circ_0087502 expression in PC tissues was shown to be strongly linked with histological grade (d), tumor stage (e), and lymph node metastasis (f). Individuals with PC (g) whose circ_0087502 expression was higher had a worse probability of surviving. (h) When compared to linear IARS, the stability of circ_0087502 was little affected by RNase R treatment. (I) circ_0087502 was detected in both the cytoplasmic and nuclear fractions of the PC cells, as determined by qRT-PCR. ***P < 0.001.

### Circ_0087502 facilitates PC cell growth, migration, invasion, and chemoresistance

**Figure 2.**
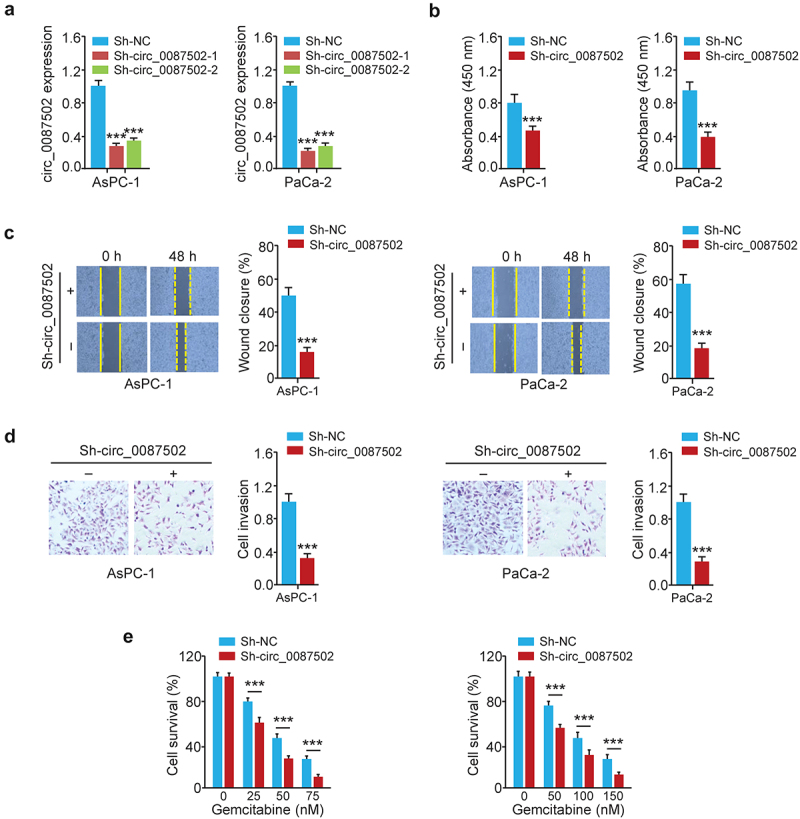
Circ_0087502 facilitates PC cell growth, migration, invasion, and chemoresistance.

**Figure 3.**
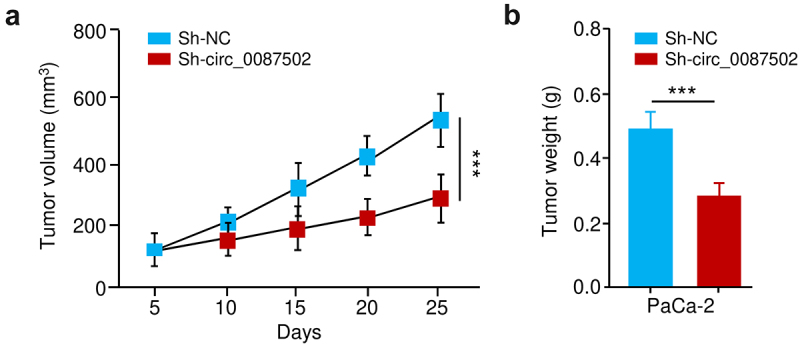
Circ_0087502 enhances PC progression in vivo.

After verifying the high expression of circ_0087502 in two PC cells (Figure 1b), we decided to analyze the biological roles of circ_0087502 using two shRNA vectors that specifically target the circ_0087502 back-splicing site. The qRT-PCR assays suggested that the expression of circ_0087502 was significantly reduced in AsPC-1 and PaCa-2 cells after transfection with the oligonucleotides (Figure 2a). Because the first shRNA, namely sh-circ_0087502–1, had higher silencing efficacy than the second shRNA, it was chosen for the downstream study (Figure 2a). CCK-8, wound-healing, transwell invasion, and drug sensitivity assay showed that downregulating circ_0087502 expression greatly reduced the capacity of AsPC-1 and PaCa-2 cells to proliferate, migrate, invade and be resistant to gemcitabine (Figure 2b–e). Furthermore, circ_0087502-silenced or control PaCa-2 cells were then subcutaneously implanted into BALB/c nude mice. Every 5 days, the tumor volume and weight of the naked mice were assessed. 25 days after the injection, the tumors were removed and weighed. Tumor xenografts revealed that in the sh-circ_0087502–1 group, the volume and weight of the generated tumors were smaller than those in the control shRNA (sh-NC) group (Figure 3a,b). These in vitro and in vivo findings collectively showed that circ_0087502, a circRNA upregulated in PC tissues, enhances PC chemoresistance and progression.

(a) Using qRT-PCR, we found that circ_0087502 expression was indeed reduced in PC cells after being knocked down by shRNAs. (b-d) In a number of experiments evaluating cell growth, wound healing, and invasion, the sh-circ_0087502 group dramatically underperformed the sh-NC group in terms of cell proliferation (B), migration (C), invasion (D), and resistance to gemcitabine (E). ***P < 0.001.

Results from tumor xenografts showed that tumors grown with sh-circ_0087502-1 shRNA were smaller (Figure 3a) and weighed (Figure 3b) less than those grown with sh-NC shRNA. ***P < 0.001.

### Circ_0087502 sponges miR-1179 and acts as a competing endogenous RNA (ceRNA)

**Figure 4.**
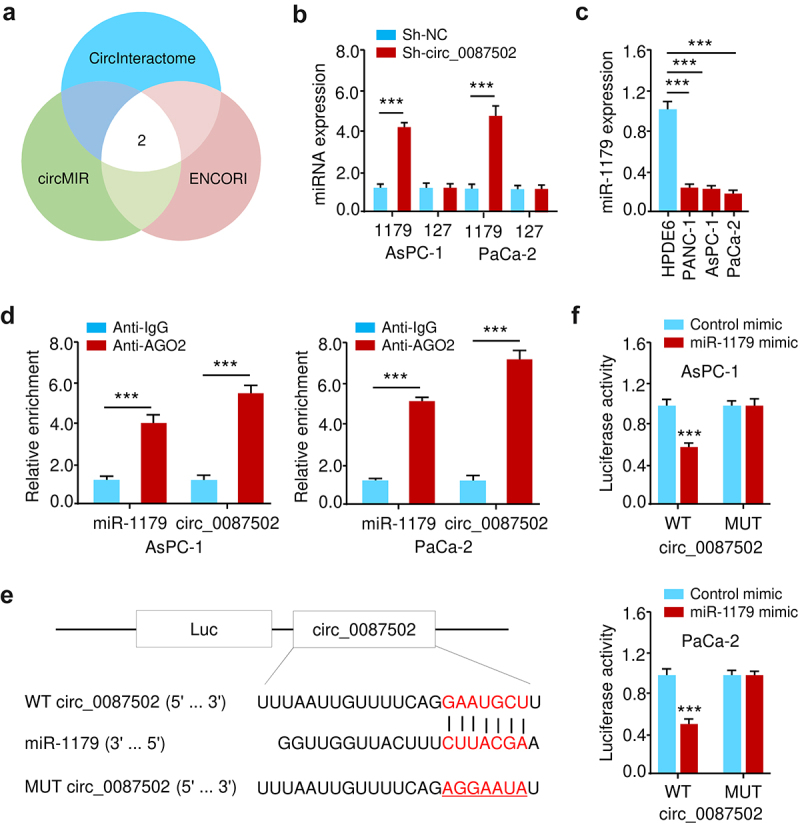
Circ_0087502 sponges miR-1179 and acts as a ceRNA.

Because of its numerous possible miRNA binding sites, circular RNA behaves as a sponge molecule in the post-transcriptional phase^5^. We hypothesized that circ_0087502 could sponge several critical miRNAs implicated in PC progression and chemoresistance. To screen the putative miRNAs for circ_0087502, we used the bioinformatics tools (circMIR, CircInteractome, and ENCORI). All three bioinformatics algorithms predicted two miRNAs (miR-1179 and miR-127) to bind circ_0087502 simultaneously (Figure 4a). Then, the levels of miR-1179 and miR-127 were examined via qRT-PCRs in AsPC-1 and PaCa-2 cells transfected with sh-circ_0087502 or sh-NC. In circ_0087502-knockdown PC cells, miR-1179 (but not miR-127) expression was shown to be increased (Figure 4b). Furthermore, we reported that miR-1179 was downregulated in PC cell lines compared to normal cells (Figure 4c). The association between miR-1179 and circ_0087502 was analyzed in the RIP assay using an Ago2 antibody or normal anti-IgG. In AsPC-1 and Paca-2 cells, miR-1179 and circ_0087502 were more abundant in the Ago2 pellet than in the IgG pellet (Figure 4d), suggesting that circ_0087502 might sponge miR-1179. The wild-type (WT) dual-luciferase reporter vector of circPPP6R3 was then designed to investigate the binding interaction between circ_0087502 and miR-1179 (Figure 4e). The dual-luciferase reporter assay revealed that miR-1179 mimics dramatically reduced the luciferase activity of circ_0087502 as compared to the control mimics group (Figure 4f). When the corresponding binding sites of miR-1179 in the circ_0087502-carrying luciferase vector were mutated (MUT), we detected no difference in the luciferase activity when miR-1179 mimics were transfected against control mimics (Figure 4f). Overall, these findings suggested that in PC cells, circ_0087502 acts as a sponge for miR-1179.

(a) Based on three online databases, two miRNAs, miR-1179 and miR-127, were identified to have probable binding sites for circ_0087502. (b) miR-1179 and miR-127 levels were measured with qRT-PCR in PC cells with circ_0087502 silencing. (c) MiR-1179 levels in PC cells when compared to those in normal cells. (d) Both miR-1179 and circ_0087502 were shown to bind to the Ago2 protein in RIP experiments. (e) Potential binding sites for miR-1179 and circ_0087502 have been predicted using online databases. (f) Circ_0087502 was shown to be able to bind to miR-1179 in a luciferase reporter activity experiment. WT, wild-type. MUT, mutation. ***P < 0.001.

### Circ_0087502 functionally interacts with miR-1179 to serve as an oncogenic circRNA

**Figure 5.**
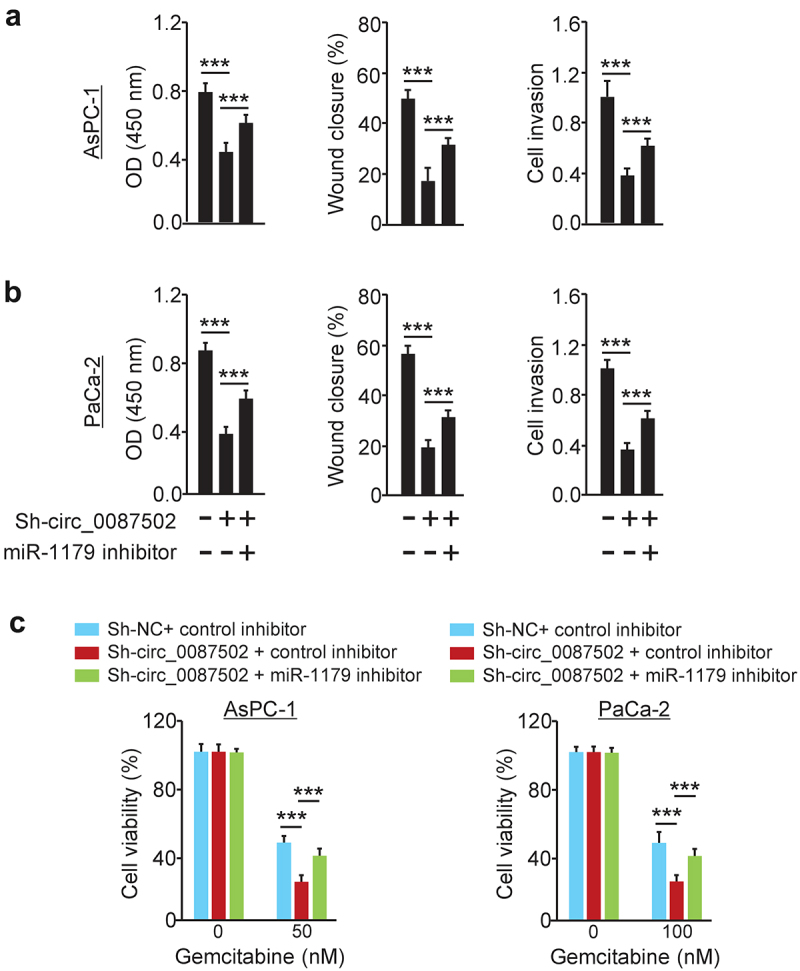
Circ_0087502 functionally interacts with miR-1179 to serve as an oncogenic circRNA.

MiR-1179 was previously found to repress the proliferation, migration, and invasion of PC cells^9^, and its levels were correlated with better PC patient survival^10^. However, the biological activities of miR-1179 in PC chemoresistance were not recognized. To check whether circ_0087502 has a regulatory effect on PC progression and chemoresistance by sponging miR-1179, we performed rescue experiments. In PC cells, we initially co-transfected sh-circ_0087502 (or sh-NC), with (or without) miR-1179 inhibitor, and conducted cell functional experiments. We confirmed that miR-1179 inhibitors had a rescue impact on the cellular proliferation, migration, invasion, and gemcitabine resistance, which were suppressed by circ_0087502 knockdown (Figure 5a–c). To summarize, these findings showed that circ_0087502 functions biologically by interacting with miR-1179 in PC cells.

Both AsPC-1 (a) and PaCa-2 (b) cell proliferation, migration, and invasion were inhibited when circ_0087502 was downregulated by shRNA, but were largely recovered after co-transfection with a miR-1179 inhibitor. (c) After treating PC cells with gemcitabine, downregulation of circ_0087502 by shRNA reduced cell viability; however, co-transfection with a miR-1179 inhibitor partly recovered cell viability. ***P < 0.001.

### MiR-1179 targets a known oncogene TGFBR2

**Figure 6.**
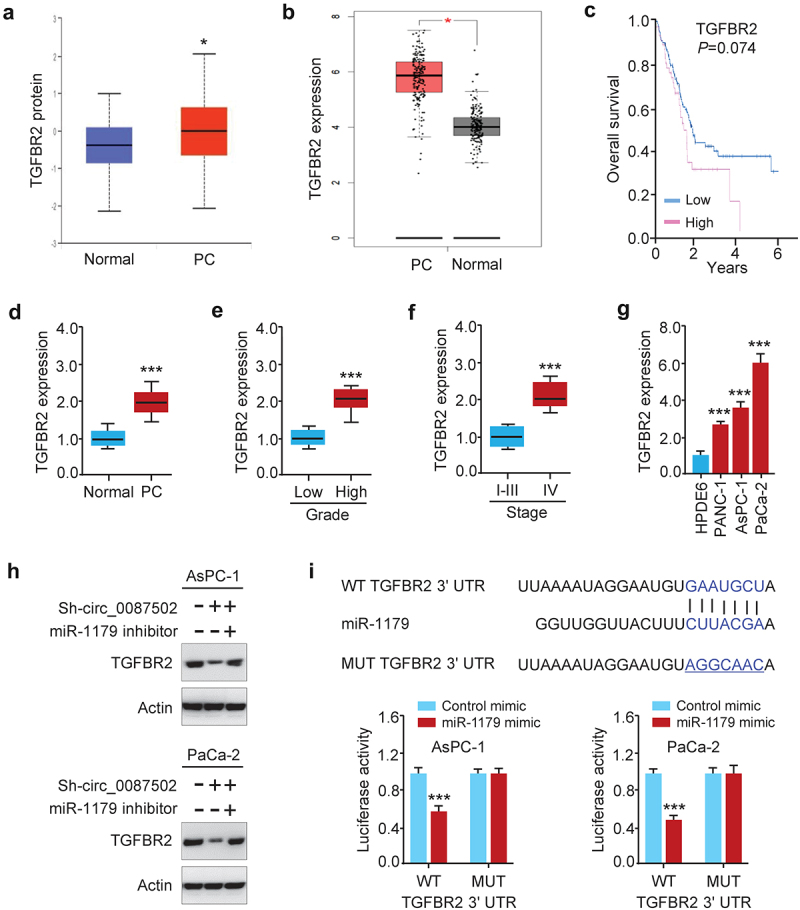
MiR-1179 targets a known oncogene TGFBR2.

MiR-1179 suppresses the proliferation, migration, and invasion of human PC cells by targeting E2F5 in prior research^9^. Nonetheless, the target genes of miR-1179 in PC cells, and whether the targets are involved in chemoresistance remain unknown. As a consequence, we used the Targetscan database to discover putative miR-1179 target genes. Although many candidate genes emerged, TGFBR2, a predicted target, stood out and was chosen for further analysis, based on previous reports showing that TGFBR2 overexpression increases PC cell proliferation, migration, invasion, and EMT^11,12^. Using the UALCAN (Figure 6a) and GEPIA databases (Figure 6b), we showed that in TCGA PC samples, TGFBR2 levels were increased in PC tissues compared to normal tissues. Also, TGFBR2 expression was shown to be linked to a poor outcome in PC patients, as demonstrated using the HPA database (Figure 6c). The expression of TGFBR2 was significantly higher in PC tissues than in the adjacent normal tissues (Figure 6d). The clinical tumor grade and tumor stage of PC patients were all positively linked with high TGFBR2 expression levels (Figure 6e, f). Moreover, TGFBR2 levels were increased in three PC cell lines than in normal cells (Figure 6g). The expression of TGFBR2 was suppressed in the group treated with both sh-circ_0087502 and control inhibitor. However, it was notably increased in the group treated with sh-circ_0087502 and the miR-1179 inhibitor, regardless of whether gemcitabine was present or not (Figures 6h and S1). These data uncovered that the expression of TGFBR2 was regulated by circ_0087502 and miR-1179. Our luciferase reporter assay demonstrated that in the wild-type (WT) TGFBR2 3´-UTR-transfecting cells, miR-1179 mimics dramatically reduced the luciferase activity (Figure 6I). Furthermore, when we altered the miR-1179 binding sites in plasmids expressing TGFBR2 3´-UTR, the mutations were able to restore the luciferase activity to a great extent, demonstrating that miR-1179 and TGFBR2 have an interaction connection.

Both AsPC-1 (A) and PaCa-2 (B) cell proliferation, migration, and invasion were inhibited when circ_0087502 was downregulated by shRNA, but were largely recovered after co-transfection with a miR-1179 inhibitor. (C) After treating PC cells with gemcitabine, downregulation of circ_0087502 by shRNA reduced cell viability; however, co-transfection with a miR-1179 inhibitor partly recovered cell viability. ***P < 0.001.

### TGFBR2 knockdown represses PC cell proliferation, migration, invasion, and chemoresistance

**Figure 7.**
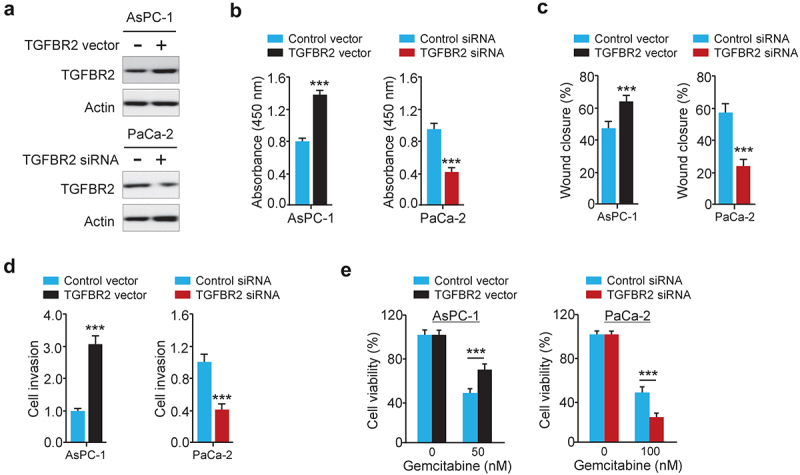
TGFBR2 knockdown represses PC cell proliferation, migration, invasion, and chemoresistance.

**Figure 8.**
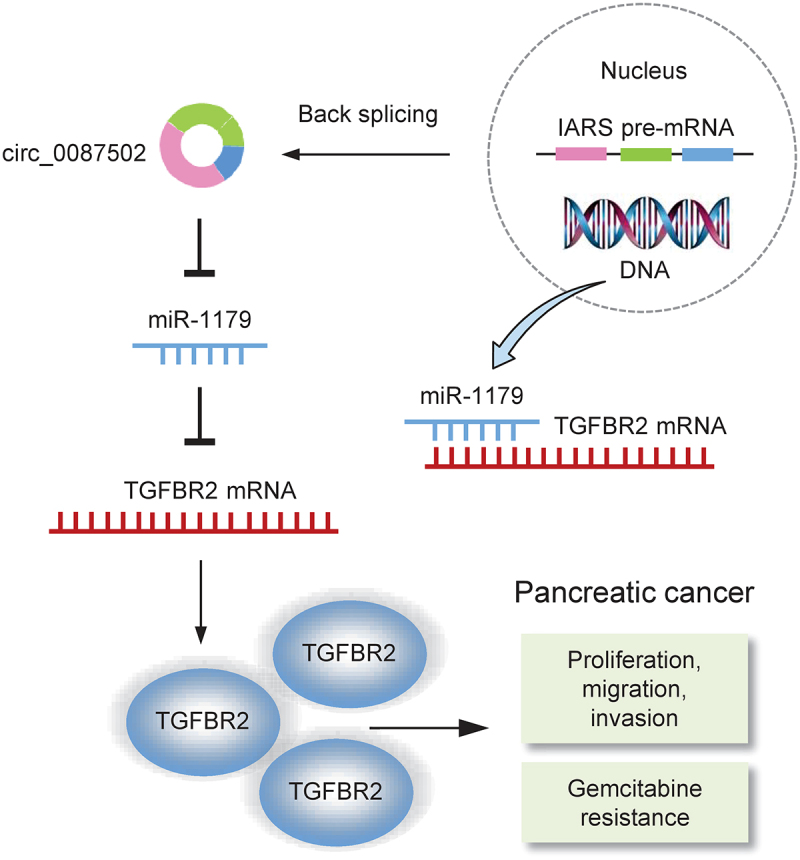
The biological activities and mechanisms of the circ_0087502/miR-1179/TGFBR2 regulatory axis in PC development and chemoresistance.

To explore the roles of TGFBR2 in PC progression and chemoresistance, we used the TGFBR2 expression vector and also constructed siRNA targeting TGFBR2. The results from the western blotting assay and cell functional experiments showed that cell proliferation, migration, invasion, as well as gemcitabine resistance, was diminished in TGFBR2-silenced PaCa-2 cells (Figure 7b–e). Additionally, overexpression of TGFBR2 in AsPC-1 cells obviously induced cell growth, migration, invasion, as well as gemcitabine resistance (Figure 7b–e). These findings suggested that circ_0087502 accelerates the development and gemcitabine resistance of PC via the miR-1179/TGFBR2 axis (Figure 8).

(a) Transfection of the TGFBR2 expression vector or TGFBR2 siRNA into PC cells led to opposite effects on TGFBR2 protein expression. (b – d) The effects of TGFBR2 expression vector and TGFBR2 siRNA transfection on PC cell proliferation (B), migration (C), invasion (D), and resistance to gemcitabine (E) were investigated. ***P < 0.001.

## Discussion

One of the most urgent and difficult concerns in modern clinical oncology is cancer cells’ potential to develop resistance to chemotherapy. The problem is most serious in PC, where tumors are unresectable in approximately 80% of patients, making radio/chemotherapy as a feasible option^1^. Even though gemcitabine-based chemotherapy has improved the prognosis of patients with PC, its effect is limited due to considerable drug resistance^3^. Drug resistance in PC has been attributed to some biological pathways. Gemcitabine can cause cancer cells to undergo apoptosis by interrupting DNA synthesis^13^. Nucleotide metabolism enzymes, the apoptotic route, ABC transporter proteins, activation of cancer stem cells, and the EMT pathway are the major molecular and cellular alterations that might lead to gemcitabine resistance^14^. In addition, Hedgehog, Wnt, and Notch, which govern embryonic development and somatic stem cells, have all been shown to be reactivated in cancer cells resistant to gemcitabine^14^. CircRNAs and miRNAs have recently been involved in the development of cancer-drug resistance in pancreatic and other cancer types, according to new research^6,7^. However, the multiple processes that cause gemcitabine resistance are mainly unclear. In the present study, we reported that circ_0087502 levels were highly elevated in PC tissues and cells and that circ_0087502 knockdown decreased the proliferation, invasion, and gemcitabine resistance of PC cells. Furthermore, circ_0087502 expression was linked to a poor clinical outcome in PC patients. Overall, this research sheds light on the mechanisms that lead to GEM resistance in PC cells, showing that circ_0087502 may be an important oncogene in PC and a promising therapeutic target.

CircRNAs, unique non-coding RNA family members, have critical functions in a variety of malignancies^4,5^. Previous studies have used circRNA sequencing to find differentially expressed circRNAs in PC and normal tissues^15,16^. A large number of circRNAs is deregulated in PC tissues^15,16^. For example, 169 differently expressed circRNA were found in PC cells compared to non-tumor human pancreatic ductal epithelial cells using circRNA sequencing, and circFOXK2 was shown to be upregulated significantly in PC cells and primary PCs^17^. Circ_0009065 was elevated in a cohort of PC patients^18^. Ectopic expression of circ_0009065 was associated with a worse outcome in patients with PC and positively connected with the tumor stage^18^. Although circ_0087502 was shown to be substantially expressed in PC tissues, its clinical significance in PC has yet to be determined. Our results suggested that circ_0087502 is correlated with poorer clinicopathological characteristics (including tumor grade, stage, and lymph node metastasis) and worse outcomes in PC patients. Thus, the presence of circ_0087502 in PC tissues might be employed as a predictive predictor of PC patients’ overall survival. Future research is needed to reveal whether circ_0087502 levels in body fluids (such as blood and urine) might be useful biomarkers for PC diagnosis and prognosis prediction.

Mounting research has suggested that circRNAs influence cellular activity as miRNA sponges^5–7^. For instance, circHIPK3 is a common circRNA generated from exon 2 of the *HIPK3* gene^18^. CircHIPK3 has been shown to respond to 9 miRNAs with 18 putative binding sites in a luciferase screening assay^18^. By directly binding to miR-124, circHIPK3 suppresses its function^18^. Moreover, according to a previous study, ciRS-7 functions as a sponge for miR-7, resulting in greater amounts of miR-7 targets, according to prior research^19^. After validating the location of circ_0087502 in PC cell lines, we utilized RIP assays to reveal that circ_0087502 and its potential target miRNA miR-1179 were enriched in the Ago2 complex. Furthermore, luciferase reporter assays were used to establish the sponging effect of circ_0087502 on miR-1179, and our results confirmed the direct binding between circ_0087502 and miR-1179. Our findings support the idea that circ_0087502 binds to miR-1179 and functions as a “miRNA sponge”^20^, allowing the development and chemoresistance of PC. Considering the fact that circRNA might have multiple miRNA-binding sites^18,19^, circ_0087502 may have an impact on a wide range of biological events in PC, probably due to the way it regulates a number of miRNAs and related gene networks. Therefore, our study did not rule out the possibility that targeting other miRNAs by circ_0087502 was responsible for circ_0087502‘s function in accelerating PC development and treatment resistance.

Previous study has revealed that MiR-1179 mediates anticancer effects in a variety of cancers^21,22^. MiR-1179 inhibited cancer cell proliferation and migration in cervical cancer cells^21^. Another research found miR-1179 to be a target of circ_0062389^22^. Furthermore, miR-1179 inhibitors were shown to be moderately effective in mitigating the effects of circ_0062389 knockdown on papillary thyroid cancer cells^22^. According to recent study^23^, miR-1179 overexpression suppressed AKT signaling activity in non-small cell lung cancer cells. They discovered that miR-1179 inhibited lung cancer cell growth and invasion^23^. Overexpression of miR-1179, which targets the E2F transcription factor 5, similarly inhibited PC cell motility and invasion^9^. The long non-coding RNA TMPO-AS1 enhanced docetaxel resistance in breast cancer cells by sponging miR-1179 and upregulating the expression of tripartite motif-containing protein 37 (TRIM37)^24^. In this work, we found that circ_0087502 sponged miR-1179, reducing its targeting function on TGFBR2, resulting in increased TGFBR2 expression, PC cell proliferation, migration, invasion, and gemcitabine resistance. The identification of the circ_0087502/miR-1179/TGFBR2 axis adds to our knowledge of the mechanisms underlying PC development and chemoresistance.

TGFBR2 was known to activate the downstream TGF-β signaling during tumorigenesis^25^. It has been demonstrated that TGFBR2 is dysregulated in a variety of human malignancies, including breast cancer^26^, colorectal cancer^27^, prostate cancer^28^, and PC^11,12^. Interestingly, TGFBR2 was identified as a direct target of miR-204 in gastric cancer cells, where miR-204 overexpression inhibited gastric cancer cell growth, invasion, and migration, and sensitized gastric cancer cells to 5-FU in vitro^29^. When TGFBR2 expression was restored in miR-204-overexpressing gastric cancer cells, they regained resistance to 5-FU treatment^29^. Although previous reports showed that TGFBR2 functioned as a key oncogene to increase PC cell proliferation, migration, invasion, and EMT^11,12^, the roles of TGFBR2 in gemcitabine sensitivity in PC are still unknown. In the current work, we reported that TGFBR2 overexpression dramatically induced proliferation, migration, invasion, and gemcitabine resistance in PC cells. Importantly, inhibiting TGFBR2 expression with siRNA significantly reduced these malignant properties. Our findings support the use of TGFBR2 as a therapeutic biomarker of PC.

In this investigation, we reported that increased expression of circ_0087502 was associated with poor prognosis in PC patients, suggesting the potential of this circRNA as a novel biomarker for PC. A potential next step would be to perform further experiments to validate this possibility in a larger cohort of PC patients. In addition to biomarker validation, it would also be valuable to explore the therapeutic potential of targeting circ_0087502 or its downstream effectors, such as miR-1179 and TGFBR2. This could involve the development of miRNA-based therapies or small-molecule inhibitors of TGFBR2 to target these molecules in PC cells and testing their efficacy in preclinical models.

Our findings show that circ_0087502 has an oncogenic role in PC development and gemcitabine resistance by acting as a miR-1179 sponge, producing the circ_0087502/miR-1179/TGFBR2 axis. These results might lead to the development of new biomarkers and a better understanding of the underlying mechanisms of PC.

## Supplementary Material

Supplemental Material

## Data Availability

All data used in this study will be available upon request to the corresponding author via email.
