# Supplementary material for: Dysregulation of the circ_0087502/miR-1179/TGFBR2 pathway supports gemcitabine resistance in pancreatic cancer
Source: Cancer Biol Ther. 2023 Oct 16;24(1):2258566. doi: 10.1080/15384047.2023.2258566 (PMC10580792; doi:10.1080/15384047.2023.2258566)
Supplement: Supplemental Material [file KCBT_A_2258566_SM1522.docx]

Supplementary information

Dysregulation of the circ_0087502/miR-1179/TGFBR2 pathway supports gemcitabine resistance in pancreatic cancer

Mingliu Chen^1†^, Xinxiu Liu^2†^, Jinpeng Lu^1†^, Haiwen Teng^1^, Chengui Yu^1^, Yingchun Liu^3*^, and Yansong Zheng^1*^

^1^Department of hepatobiliary and pancreatic surgery, The First Affiliated Hospital of Fujian Medical University, No. 20, Chazhong Road, Fuzhou City, Fujian Province 350004, China

^2^Department of ultrasound, The First Affiliated Hospital of Fujian Medical University, No. 20, Chazhong Road, Fuzhou City, Fujian Province 350004, China

^3^Department of Cell Biology and Genetics, Fujian Medical University, No. 20, Chazhong Road, Fuzhou City, Fujian Province 350004, China

^†^Contributed equally

*Correspondence: Dr. **Yansong Zheng**, Department of hepatobiliary and pancreatic surgery, The First Affiliated Hospital of Fujian Medical University, No. 20, Chazhong Road, Fuzhou City, Fujian Province 350004, China. Fax number: +86-591-87982772, Email: [qftes325@163.com](mailto:qftes325@163.com) ; Dr. **Yingchun Liu**, Department of Cell Biology and Genetics, Fujian Medical University, No. 20, Chazhong Road, Fuzhou City, Fujian Province 350004, China. Email: [lycmellisa@126.com](mailto:lycmellisa@126.com)

**FIGURE LEGEND**


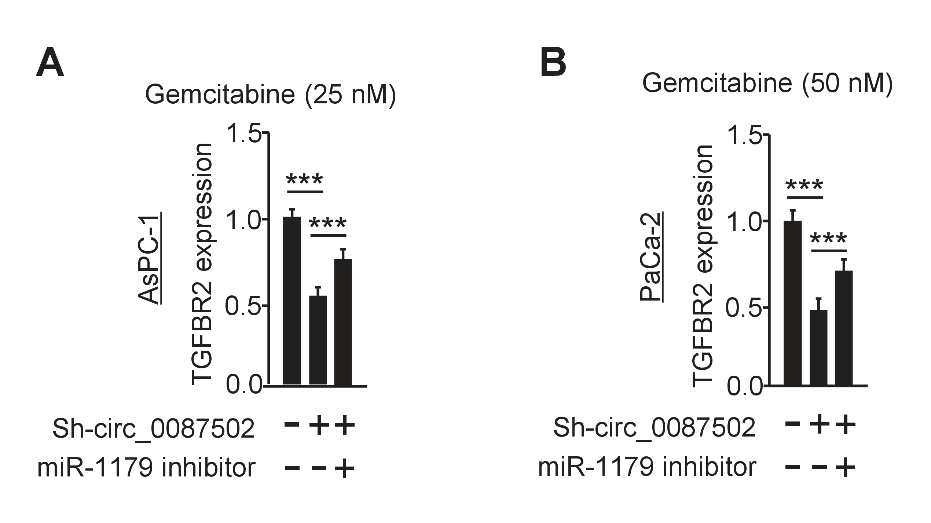


**Fig. S1: TGFBR2 expression in circ_0087502-silenced PC cells under chemotherapy was examined by qRT-PCRs.**  AsPC-1 (A) and PaCa-2 (B) cells were transfected as indicated and then treated with Gemcitabine for 48 h. The mRNA expression of TGFBR2 expression was examined by qRT-PCRs. ****P* < 0.001.
